# Supplementary material for: Workflow enabling deepscale immunopeptidome, proteome, ubiquitylome, phosphoproteome, and acetylome analyses of sample-limited tissues
Source: Nat Commun. 2023 Apr 3;14:1851. doi: 10.1038/s41467-023-37547-0 (PMC10070353; doi:10.1038/s41467-023-37547-0)
Supplement: Supplementary file 1 — Supplementary Information [file 41467_2023_37547_MOESM1_ESM.pdf]

**Supplementary Information**

Workflow enabling deepscale immunopeptidome, proteome, ubiquitylome, phosphoproteome and acetylome analyses of sample-limited tissues

**Supplementary Figure Legends:**

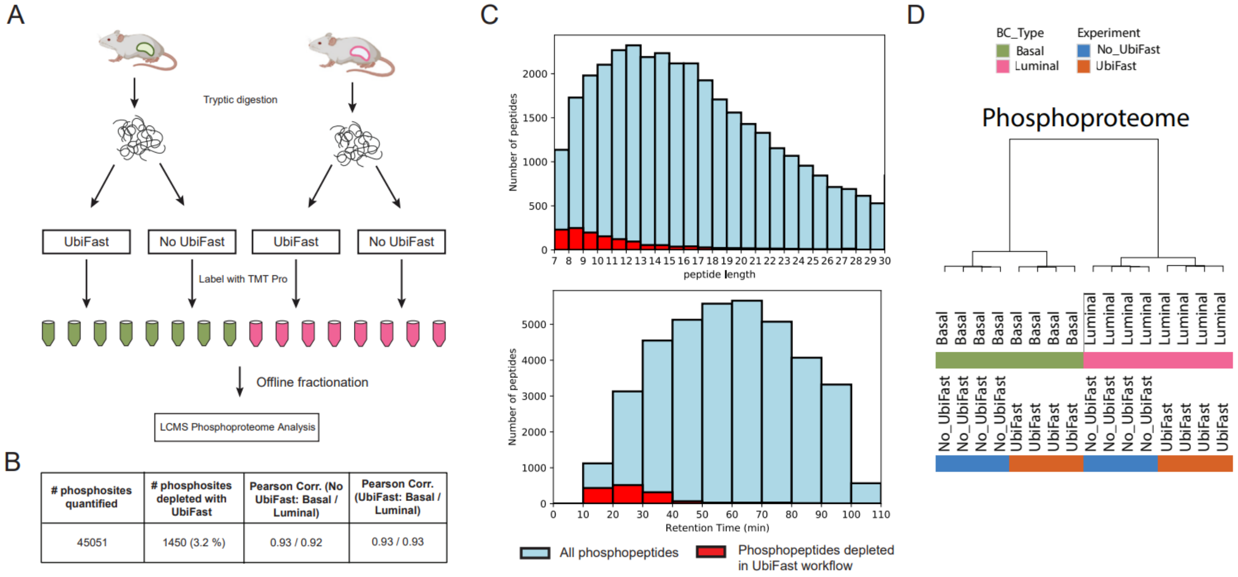


| # phosphosites quantified | # phosphosites depleted with UbiFast | Pearson Corr. (No UbiFast: Basal / Luminal) | Pearson Corr. (UbiFast: Basal / Luminal) |
|---|---|---|---|
| 45051 | 1450 (3.2 %) | 0.93 / 0.92 | 0.93 / 0.93 |

**Supplementary Figure 1: Effect of UbiFast on serially enriched phosphoproteomes. A**) TMTpro16 plex experimental design used to analyze phosphopeptides from PDX samples (CompRef) +/- UbiFast. **B**) Table containing the numbers of significantly changing human phosphorylation sites across conditions (adj. p-value <0.05 and log fold change >2) and Pearson correlation of replicates. Significantly changing sites determined using moderated two sample t-test. **C**) Histograms showing peptide length distribution of all phosphopeptides and phosphopeptides depleted after UbiFast (top) and HPLC retention time distribution of all phosphopeptides and phosphopeptides depleted after UbiFast (bottom). **D**) Dendrogram showing unsupervised hierarchical clustering of phosphoproteome data by breast cancer subtype. Source data for B and C are provided as a **Source Data** file.

A.

| sample_annotation | DPA1-1 | DPA1-2 | DPB1-1 | DPB1-2 | DQA1-1 | DQA1-2 | DQB1-1 | DQB1-2 | DRA1-1 |
|---|---|---|---|---|---|---|---|---|---|
| C3L-01632 | DPA1*01:03:01 | DPA1*01:03:01 | DPB1*02:01:02 | DPB1*04:01:01 | DQA1*05:05:01 | DQA1*01:02:01 | DQB1*06:04:01 | DQB1*03:01:01 | DRA*01:01:01 |
| C3L-02549 | DPA1*01:03:01 | DPA1*01:03:01 | DPB1*15:01:01 | DPB1*04:01:01 | DQA1*01:03:01 | DQA1*01:02:01 | DQB1*06:01:01 | DQB1*06:02:01 | DRA*01:02:02 |
| C3N-00169 | DPA1*04:01:01 | DPA1*02:02:02 | DPB1*107:01 | DPB1*05:01:01 | DQA1*03:02:01 | DQA1*01:02:02 | DQB1*05:02:01 | DQB1*03:03:02 | DRA*01:01:01 |
| C3N-00199 | DPA1*02:02:02 | DPA1*01:03:01 | DPB1*01:01:01 | DPB1*04:01:01 | DQA1*02:01:01 | DQA1*01:02:02 | DQB1*02:02:01 | DQB1*05:02:01 | DRA*01:01:01 |
| C3N-00547 | DPA1*02:02:02 | DPA1*01:03:01 | DPB1*05:01:01 | DPB1*02:01:02 | DQA1*06:01:01 | DQA1*03:01:01 | DQB1*03:02:01 | DQB1*03:01:01 | DRA*01:01:01 |
| C3N-00579 | DPA1*04:01:01 | DPA1*01:03:01 | DPB1*107:01 | DPB1*03:01:01 | DQA1*06:01:01 | DQA1*03:02:01 | DQB1*03:01:01 | DQB1*03:03:02 | DRA*01:01:01 |
| C3N-01016 | DPA1*01:03:01 | DPA1*01:03:01 | DPB1*04:01:01 | DPB1*04:01:01 | DQA1*05:01:01 | DQA1*03:03:01 | DQB1*04:01:01 | DQB1*02:01:01 | DRA*01:01:01 |
| C3N-01024 | DPA1*02:02:02 | DPA1*02:02:02 | DPB1*05:01:01 | DPB1*05:01:01 | DQA1*06:01:01 | DQA1*06:01:01 | DQB1*03:116 | DQB1*03:01:01 | DRA*01:02:02 |
| C3N-01416 | DPA1*04:01:01 | DPA1*02:01:01 | DPB1*107:01 | DPB1*13:01:01 | DQA1*06:01:01 | DQA1*03:02:01 | DQB1*03:03:02 | DQB1*03:01:01 | DRA*01:01:01 |
| C3N-02145 | DPA1*02:02:02 | DPA1*02:02:02 | DPB1*05:01:01 | DPB1*05:01:01 | DQA1*06:01:01 | DQA1*05:03:01 | DQB1*03:01:01 | DQB1*03:116 | DRA*01:01:01 |

| sample_annotation | DRA1-2 | DRB1-1 | DRB1-2 | DRB3-1 | DRB3-2 | DRB4-1 | DRB4-2 | DRB5-1 | DRB5-2 |
|---|---|---|---|---|---|---|---|---|---|
| C3L-01632 | DRA*01:02:02 | DRB1*11:01:01 | DRB1*13:02:01 | DRB3*03:01:01 | DRB3*02:02:01 | NA | NA | NA | NA |
| C3L-02549 | DRA*01:02:03 | DRB1*15:02:01 | DRB1*15:01:01 | NA | NA | NA | NA | DRB5*01:01:01 | DRB5*01:02:01 |
| C3N-00169 | DRA*01:01:01 | DRB1*16:02:01 | DRB1*09:01:02 | NA | NA | NA | DRB4*01:03 | NA | DRB5*01:01:01 |
| C3N-00199 | DRA*01:01:01 | DRB1*16:01:01 | DRB1*07:01:01 | NA | NA | NA | DRB4*01:03 | NA | DRB5*02:02:01 |
| C3N-00547 | DRA*01:02:02 | DRB1*12:02:01 | DRB1*04:04:01 | NA | DRB3*03:01:03 | NA | DRB4*01:03 | NA | NA |
| C3N-00579 | DRA*01:02:02 | DRB1*12:02:01 | DRB1*09:01:02 | NA | DRB3*03:01:03 | NA | DRB4*01:03 | NA | NA |
| C3N-01016 | DRA*01:01:01 | DRB1*04:05:01 | DRB1*03:01:01 | DRB3*02:02:01 | NA | DRB4*01:03 | NA | NA | NA |
| C3N-01024 | DRA*01:02:02 | DRB1*12:02:01 | DRB1*12:02:01 | DRB3*03:01:03 | DRB3*03:01:03 | NA | NA | NA | NA |
| C3N-01416 | DRA*01:02:02 | DRB1*12:02:01 | DRB1*09:01:02 | NA | DRB3*03:01:03 | NA | DRB4*01:03 | NA | NA |
| C3N-02145 | DRA*01:02:02 | DRB1*12:02:01 | DRB1*13:12:01 | DRB3*03:01:03 | DRB3*02:02:01 | NA | NA | NA | NA |

B.

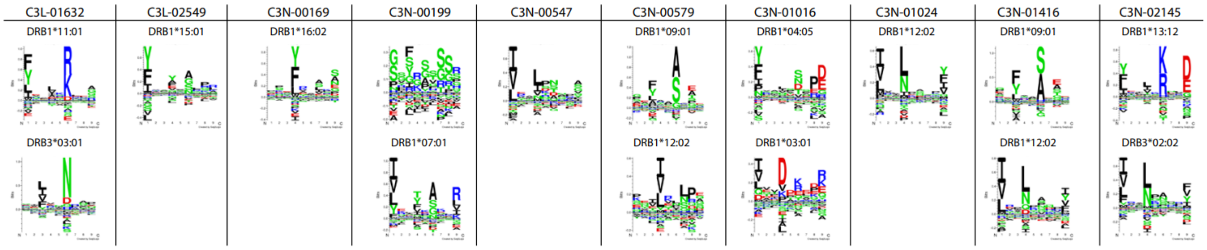


**Supplementary Figure 2: HLA-II alleles and corresponding peptide binding motifs identified from LUAD immunopeptidomes. A**) HLA-II alleles expressed by the LUAD patient cohort that were determined from RNA-Seq data using arcasHLA[4]. HLA-DRB4 alleles (red) were imputed from known genomic linkages[5,6] because no determinations were made for these alleles by arcasHLA. **B**) HLA-II binding motifs that were found using Gibbs Cluster[7] and could manually be assigned to patient HLA-II alleles.

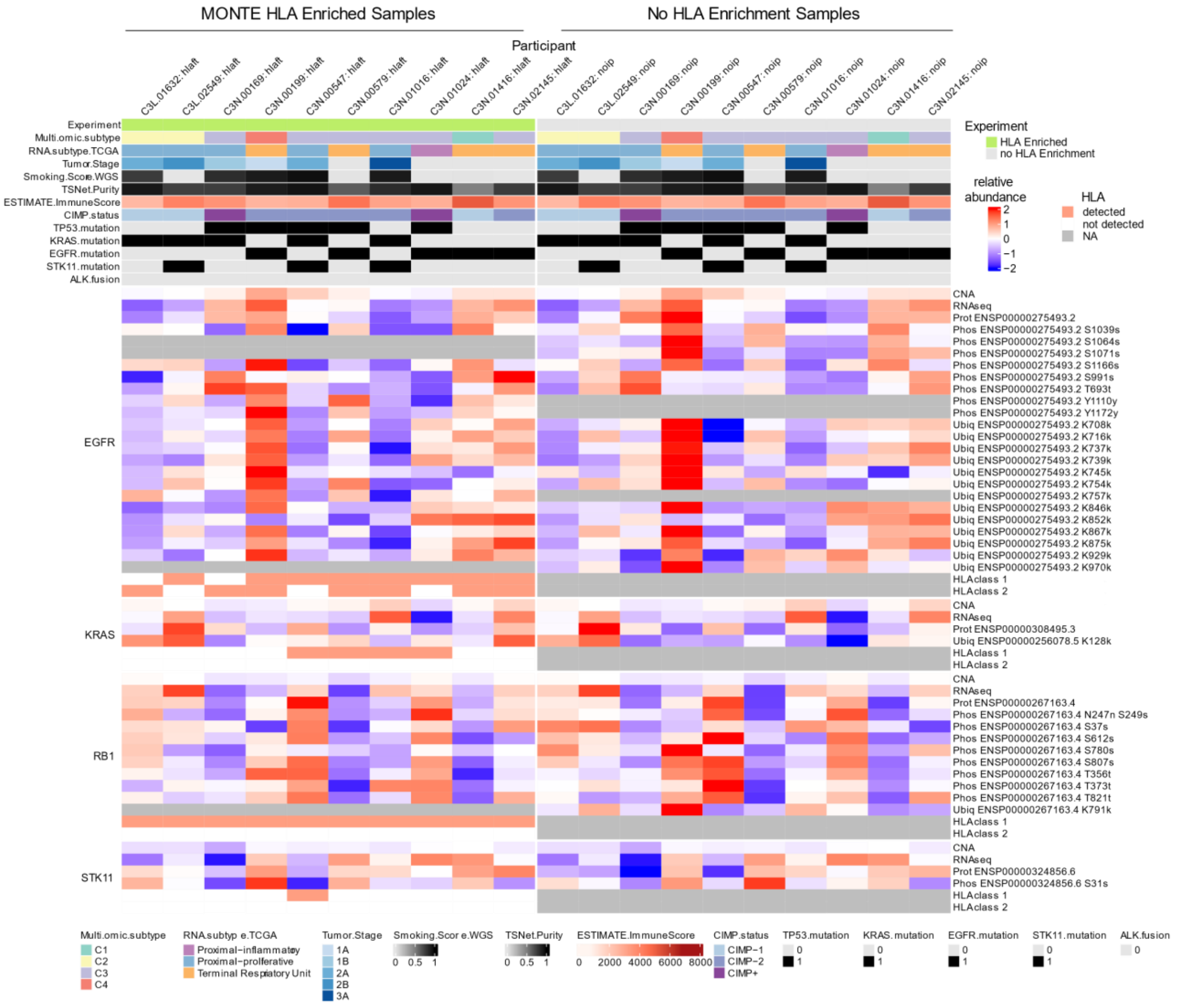


**Supplementary Figure 3: Multiomic data visualization of oncogenic and tumor suppressor proteins in the LUAD MONTE head-to-head experiment**. The heatmap depicts multi-ome data from the MONTE workflow ("HLA Enriched"; green) and the serial multi-omic enrichment workflow ("no HLA Enrichment"; gray). CNA and RNA-Seq are copied from the LUAD 2020 discovery dataset[8]. HLA tracks show whether at least one class I or class II peptide was detected in the MONTE workflow. For other data types the heatmap depicts abundances observed in tumor tissue relative to normal adjacent tissue (NAT) previously reported[8]. These data were visualized using the data viewing tool available at https://proteomics.broadapps.org/CPTAC-MONTE2022/.

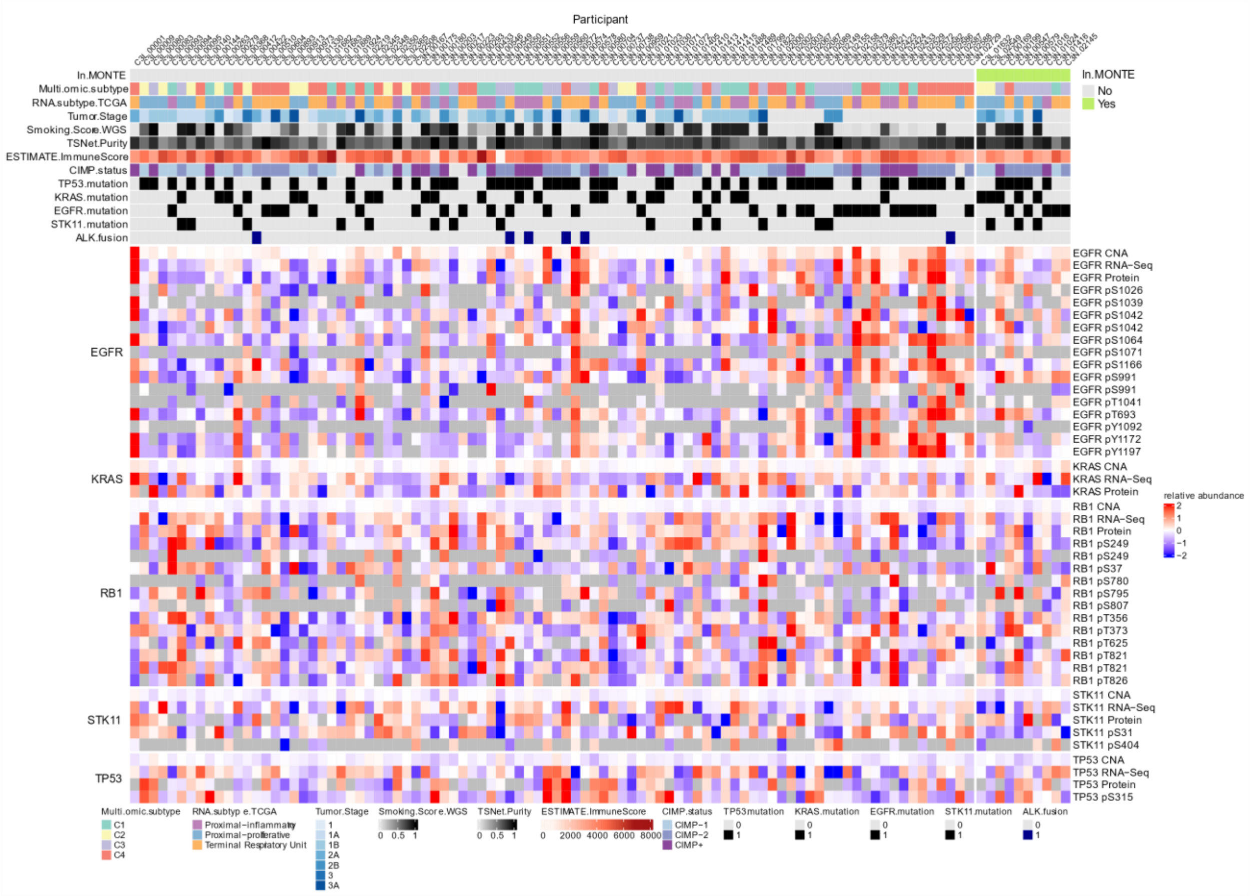


**Supplementary Figure 4: Multiomic data visualization of oncogenic and tumor suppressor proteins in a LUAD Discovery Dataset**. The heatmap depicts multi-ome data from the published LUAD discovery dataset[8] that contains the 10 LUAD samples profiled with the MONTE workflow (green). Copy number aberrations are relative to matching normal blood samples and are on log2(CNA)-1 scale. For other data types, the heatmap depicts abundances observed in tumor tissue relative to normal adjacent tissue (NAT). These data were visualized using the data viewing tool available at https://proteomics.broadapps.org/CPTAC-MONTE2022/.

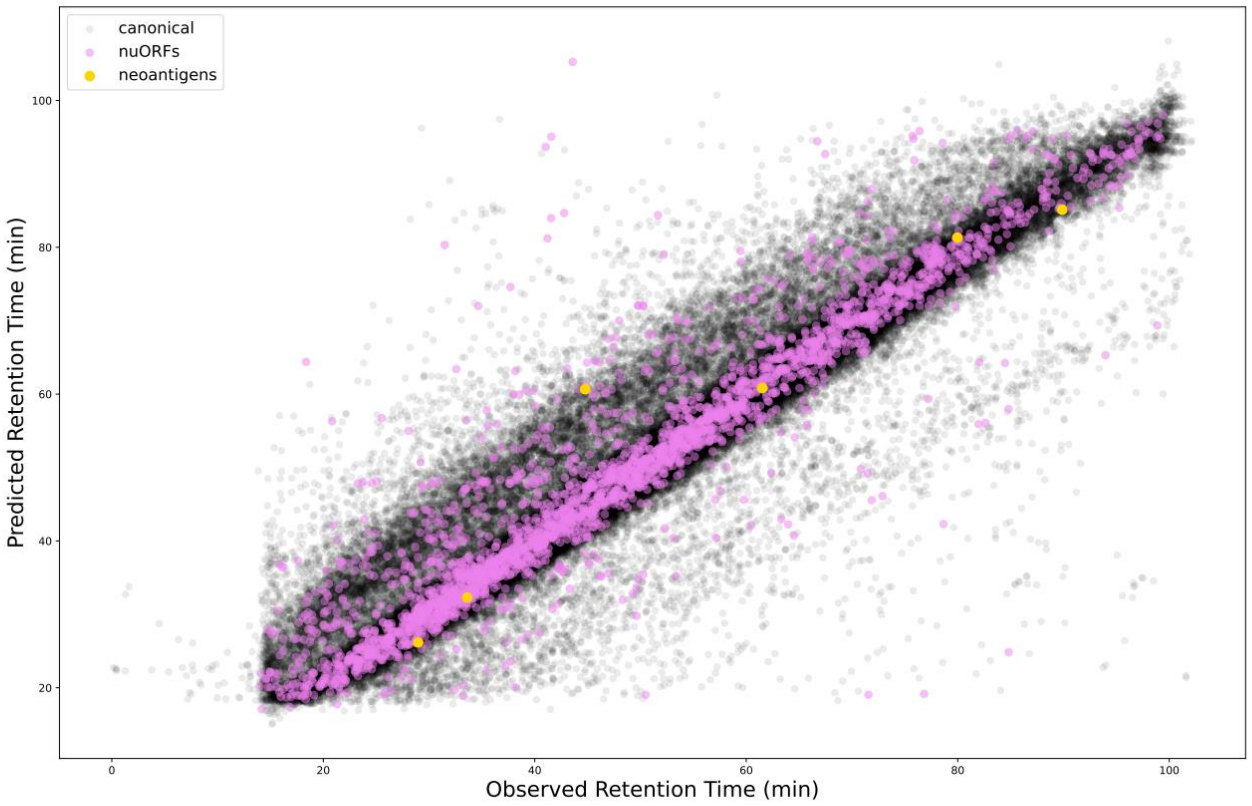


**Supplementary Figure 5: Scatter plot of the DeepLC[9]-predicted retention times vs. experimentally observed retention times of canonical, nuORF-derived, and neoantigen HLA-I peptides across the 10 LUAD immunopeptidomes**. Of the observed HLA-I peptides, 15% of total peptides have delta<1%, 61% have delta<5%, 80% have delta<10%, 88% have delta<20%, suggesting that many of the peptides appear to have the expected retention time, which further supports their sequence identifications. Source data are provided as a **Source Data** file.

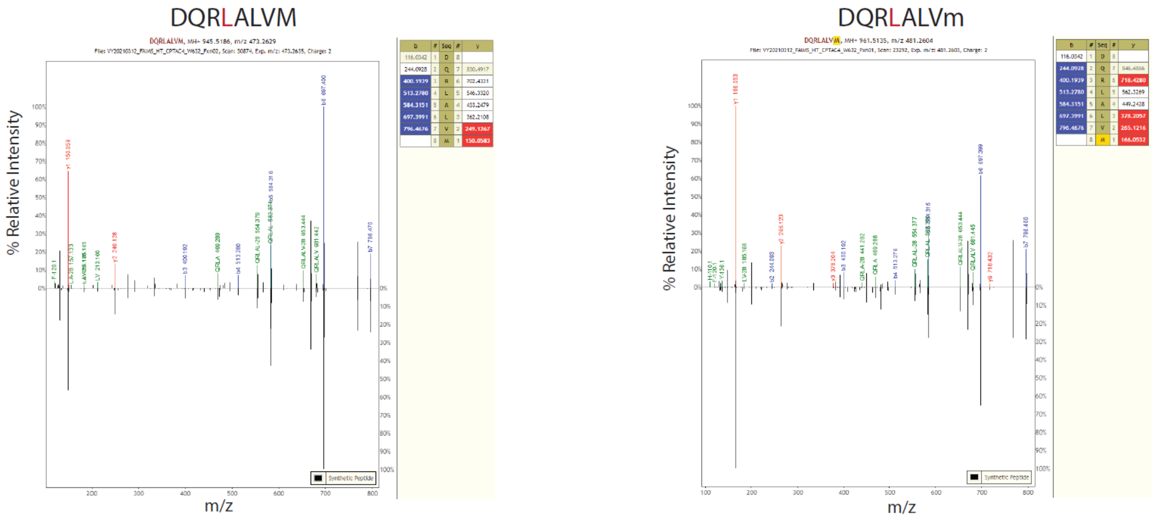

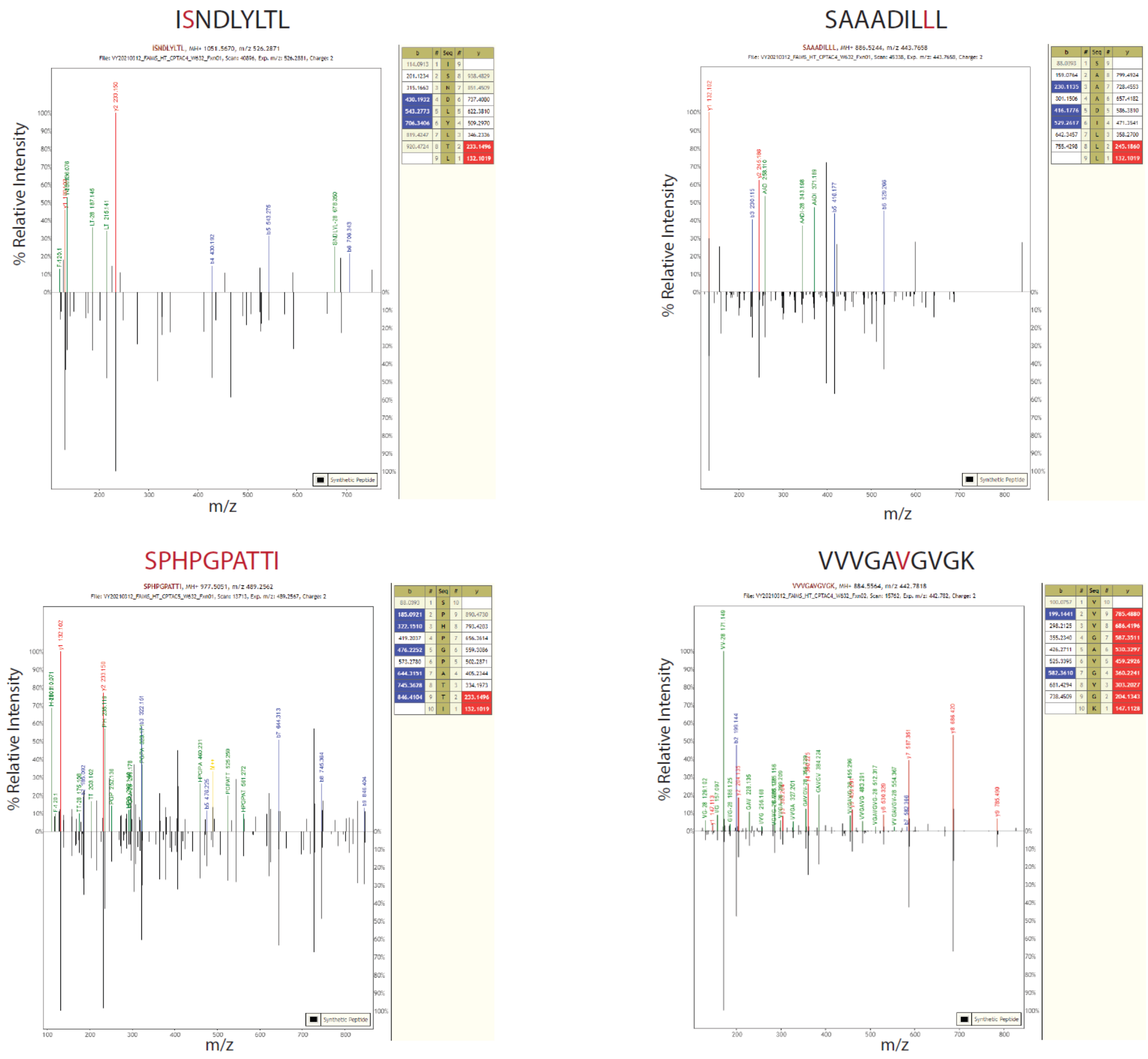


**Supplementary Figure 6: Mirror plots comparing experimentally observed and synthetic peptide MS/MS spectra of neoantigens reported in Figure 5D**. The most highly correlated synthetic spectrum for each neoantigen peptide is shown with no peak filtering applied. Leucine (L) and Isoleucine (I) cannot be distinguished by the MS instrumentation employed.

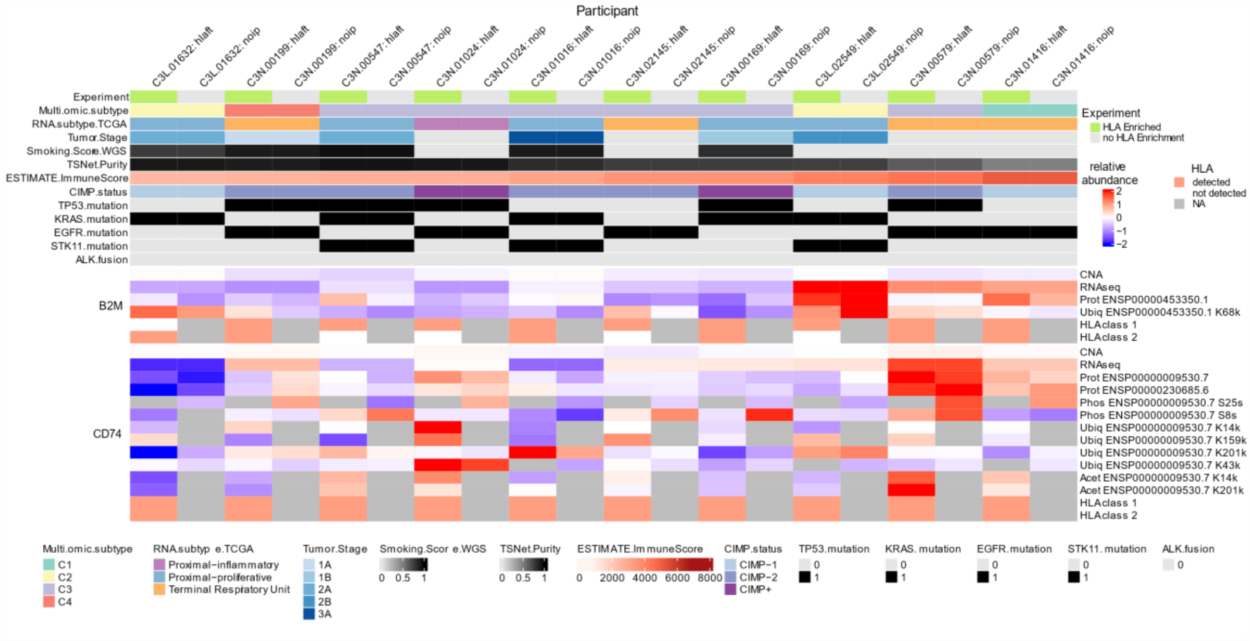


**Supplementary Figure 7: Multiomic data visualization of B2M and CD74.**The heatmap depicts multi-ome data from the MONTE workflow ("HLA Enriched"; green) and the serial multi-omic enrichment workflow ("no HLA Enrichment"; gray). CNA and RNA-Seq are copied from the LUAD 2020 discovery dataset. HLA tracks show whether at least one class I or class II peptide was detected in the MONTE workflow. For other data types, the heatmap depicts abundances observed in tumor tissue relative to normal adjacent tissue (NAT) previously reported[8]. These data were visualized using the data viewing tool https://proteomics.broadapps.org/CPTAC-MONTE2022/.

## Supplementary References

1. Almeida, L. G. *et al.* CTdatabase: a knowledge-base of high-throughput and curated data on cancer-testis antigens. *Nucleic Acids Res.* **37**, D816–9 (2009).

2. Djureinovic, D. *et al.* Profiling cancer testis antigens in non-small-cell lung cancer. *JCI Insight* **1**, e86837 (2016).

3. Ouspenskaia, T. *et al.* Unannotated proteins expand the MHC-I-restricted immunopeptidome in cancer. *Nat. Biotechnol.* (2021) doi:10.1038/s41587-021-01021-3.

4. Orenbuch, R. *et al.* arcasHLA: high-resolution HLA typing from RNAseq. *Bioinformatics* **36**, 33–40 (2020).

5. Zhao, L. P. *et al.* Next-Generation Sequencing Reveals That HLA-DRB3, -DRB4, and -DRB5 May Be Associated With Islet Autoantibodies and Risk for Childhood Type 1

Diabetes. *Diabetes* vol. 65 710–718 Preprint at https://doi.org/10.2337/db15-1115 (2016).

6. Ozaki, Y. *et al.* HLA-DRB1, -DRB3, -DRB4 and -DRB5 genotyping at a super-high resolution level by long range PCR and high-throughput sequencing. *Tissue Antigens* vol. 83 10–16 Preprint at https://doi.org/10.1111/tan.12258 (2014).
7. Andreatta, M., Alvarez, B. & Nielsen, M. GibbsCluster: unsupervised clustering and alignment of peptide sequences. *Nucleic Acids Res.* **45**, W458–W463 (2017).
8. Gillette, M. A. *et al.* Proteogenomic Characterization Reveals Therapeutic Vulnerabilities in Lung Adenocarcinoma. *Cell* **182**, 200–225.e35 (2020).
9. Bouwmeester, R., Gabriels, R., Hulstaert, N., Martens, L. & Degroeve, S. DeepLC can predict retention times for peptides that carry as-yet unseen modifications. *Nat. Methods* **18**, 1363–1369 (2021).
