# Supplementary material for: Workflow enabling deepscale immunopeptidome, proteome, ubiquitylome, phosphoproteome, and acetylome analyses of sample-limited tissues
Source: Nat Commun. 2023 Apr 3;14:1851. doi: 10.1038/s41467-023-37547-0 (PMC10070353; doi:10.1038/s41467-023-37547-0)
Supplement: Supplementary file 3 — Description of additional supplementary files [file 41467_2023_37547_MOESM3_ESM.docx]

**Description of Additional Supplementary Files**

Workflow enabling deepscale immunopeptidome, proteome, ubiquitylome, phosphoproteome and acetylome analyses of sample-limited tissues

**Supplementary Data 1:** Data related to MONTE HLA Immunopeptidome Optimizations. Average HLA peptide counts and average proteome protein counts are shown across several A375 experiments that were used to optimize parameters of the MONTE workflow. The tabs include: Proteome method (Urea vs. S-trap vs. chloroform-methanol crash comparison with and without DUB inhibitors), Acetyl inhibitor (HLA peptide counts with and without the addition of acetylase inhibitors), DUB inhibitor (HLA peptide counts with and without the addition of the deubiquitinase inhibitor), and HLA-II antibody ratios (HLA peptide counts with different ratios of DR, DP, and DQ antibodies).

**Supplementary Data 2:** Comparison of results for serial proteome, phosphoproteome, and acetylome versus initial UbiFast enrichment followed by serial enrichment of these three ‘omes. Patient-derived xenograft samples of luminal and basal breast cancer (referred to as Comparative Reference Tissue, or CompRef) were used in this comparison. Adjusted p-values were determined from using a moderated two sample t-test. GSEA results for proteome, phosphoproteome, and acetylome are included in addition to PTM-SEA results. The “Description” tab includes descriptions of all column headers.

**Supplementary Data 3:** Phosphoproteome results with and without UbiFast in a single TMT 16-plex. The “Description” tab includes descriptions of all column headers. Adjusted p-values were determined from using a moderated two sample t-test. Associated with **Supplementary Figure 1**.

**Supplementary Data 4:** LUAD tumor characteristics of samples profiled using the MONTE workflow. A description of the ten LUAD patient tumors characterized using the MONTE workflow.

**Supplementary Data 5:** HLA immunopeptidome results for Comparative Reference Tissue (CompRef) samples. A summary of total HLA-I and HLA-II peptides isolated from breast cancer patient-derived xenograft (PDX) models, representing Basal (P93; WHIM2) and Luminal (P94; WHIM16) subtypes of breast cancer.

**Supplementary Data 6:** Comparison of experimental conditions and effects of lysis buffer conditions and sample preparation steps on label-free A375 acetylome coverage. The different conditions used to test how native lysis buffer conditions, HDAC inhibitors, and empty Protein G sepharose bead preclear impacted acetylpeptide immunoenrichment are shown. Variables evaluated include different lysis buffer bases (native vs. SDS), the inclusion of HLA enrichment, timing, temperature, lysis buffer additives, the inclusion of a bead-based preclear step prior to digestion, and the inclusion of HDAC inhibitors in the lysis buffer. The resulting acetylpeptide (AcK) enrichment specificities (% spectra containing AcK) and total acetyl peptides (distinct peptides containing AcK) are shown for each sample and associated condition. The average number of distinct AcK peptides is also reported in terms of the major variables being tested.

**Supplementary Data 7:** LUAD Proteome and PTM-omes. A table describing all proteins and PTM sites detected and quantified by TMT ratios from the LUAD MONTE ‘omes. There are individual tabs for ubiquitylome, proteome, phosphoproteome, and acetylome. There also tabs for additional subsets extracted from the individual ‘omes with further subset-specific FDR filtering (**see Methods**), including: the subset of somatic variant-containing peptides detected in the proteome and the subsets of nuORFs detected in the ubiquitylome, phosphoproteome, and acetylome.

**Supplementary Data 8:** HLA peptides derived from known oncogenic and tumor suppressor genes in LUAD immunopeptidomes. HLA-I and HLA-II peptides derived from wild-type EGFR (8/10), KRAS (3/10), RB1 (10/10), TP53 (9/10), and STK11 (1/10) are shown as binary values for each unique peptide identified across the LUAD immunopeptidomes.

**Supplementary Data 9:** LUAD Immunopeptidome detection of canonical peptides and CTAs, neoantigens, nuORF-derived peptides, and PTM-containing peptides. A table describing all detected HLA peptides including those containing mutations, those mapping to CT antigens, those mapping to nuORFs, and those containing phosphorylation or acetyl modifications. The canonical_HLA-I_LUAD tab reports all human HLA-I peptides and the canonical_HLA-II_LUAD tab reports all human HLA-II peptides. The neoantigen tab displays all detected neoantigens. The HLAIHLAII_CTantigen tab contains HLA-I and HLA-II peptides derived from CT antigen source proteins found in the CTdatabase[^1^](https://paperpile.com/c/RVgahZ/kwI10). HLAIHLAII_Lung_CTantigens consists of peptides derived from lung-specific CT antigens[^2^](https://paperpile.com/c/RVgahZ/eregq). HLA-I nuORFs and HLA-II nuORFs list all HLA-I and HLA-II peptides that are mapped only to nuORF_human proteins[^3^](https://paperpile.com/c/RVgahZ/7UYwc). HLA-I_phospho_8to11mers and HLA-II_phospho are all HLA peptides containing a phospho modification. HLA-I_acetyl_8to11mers and HLA-II_acetyl are all HLA peptides containing an acetyl modification. All data except HLA-II nuORF data had tryptic contaminants and duplicates removed. HLA-II nuORF data did not have tryptics removed because a majority of typtics and were in nested sets, which are characteristic of HLA-II data. Data was additionally filtered to contain peptides derived only from Human/nuORF species with the exception of the canonical HLA tabs, which contain only peptides that map to human source proteins, and the nuORF tabs, which contain only peptides mapped uniquely to nuORF source proteins. HLA-I data was additionally filtered to contain only 8–12mers (canonical HLA-I, neoantigens, CT antigens, and nuORFs) or 8–11mers (phospho, acetyl). nuORFs, phospho peptides, and acetyl peptides were subjected to additional spectral quality filtering to decrease the number of false positives.
