# Supplementary material for: Workflow enabling deepscale immunopeptidome, proteome, ubiquitylome, phosphoproteome, and acetylome analyses of sample-limited tissues
Source: Nat Commun. 2023 Apr 3;14:1851. doi: 10.1038/s41467-023-37547-0 (PMC10070353; doi:10.1038/s41467-023-37547-0)
Supplement: Supplementary file 13 — Reporting Summary [file 41467_2023_37547_MOESM13_ESM.pdf]

nature portfolio

Corresponding author(s): Jennifer G. Abelin, Namrata D. Udeshi, Steven A. Carr

Last updated by author(s): 3/17/2023

Reporting Summary

Nature Portfolio wishes to improve the reproducibility of the work that we publish. This form provides structure for consistency and transparency in reporting. For further information on Nature Portfolio policies, see our Editorial Policies and the Editorial Policy Checklist.

Statistics

For all statistical analyses, confirm that the following items are present in the figure legend, table legend, main text, or Methods section.

n/a	Confirmed	
☐	☒	The exact sample size (*n*) for each experimental group/condition, given as a discrete number and unit of measurement
☐	☒	A statement on whether measurements were taken from distinct samples or whether the same sample was measured repeatedly
☐	☒	The statistical test(s) used AND whether they are one- or two-sided<br>*Only common tests should be described solely by name; describe more complex techniques in the Methods section.*
☒	☐	A description of all covariates tested
☐	☒	A description of any assumptions or corrections, such as tests of normality and adjustment for multiple comparisons
☐	☒	A full description of the statistical parameters including central tendency (e.g. means) or other basic estimates (e.g. regression coefficient) AND variation (e.g. standard deviation) or associated estimates of uncertainty (e.g. confidence intervals)
☐	☒	For null hypothesis testing, the test statistic (e.g. *F*, *t*, *r*) with confidence intervals, effect sizes, degrees of freedom and *P* value noted<br>*Give P values as exact values whenever suitable.*
☒	☐	For Bayesian analysis, information on the choice of priors and Markov chain Monte Carlo settings
☐	☒	For hierarchical and complex designs, identification of the appropriate level for tests and full reporting of outcomes
☐	☒	Estimates of effect sizes (e.g. Cohen's *d*, Pearson's *r*), indicating how they were calculated

Our web collection on statistics for biologists contains articles on many of the points above.

Software and code

Policy information about availability of computer code

Data collection	Xcalibur 4.0
Data analysis	Protigy, v0.9.1.3, Broad Institute, https://github.com/broadinstitute/protigy, Spectrum Mill v 7.08 (proteomics.broadinstitute.org), PTM Signature Enrichment Analysis (PTM-SEA) https://github.com/broadinstitute/ssGSEA2.0

For manuscripts utilizing custom algorithms or software that are central to the research but not yet described in published literature, software must be made available to editors and reviewers. We strongly encourage code deposition in a community repository (e.g. GitHub). See the Nature Portfolio guidelines for submitting code & software for further information.

Data

Policy information about availability of data

All manuscripts must include a data availability statement. This statement should provide the following information, where applicable:

- Accession codes, unique identifiers, or web links for publicly available datasets
- A description of any restrictions on data availability
- For clinical datasets or third party data, please ensure that the statement adheres to our policy

For the LUAD and A375 samples, the base proteome consisted of the human reference proteome Gencode 34 (ftp.ebi.ac.uk/pub/databases/gencode/Gencode_human/release_34/) with 47,429 non-redundant protein coding transcript biotypes mapped to the human reference genome GRCh38, 602 common laboratory contaminants, 2043 curated smORFs (lncRNA and uORFs), 237,427 novel unannotated ORFs (nuORFs) supported by ribosomal profiling nuORF DB v1.052, and 4,167 TCGA shared mutations from 26 tumor types (https://www.cancer.gov/tcga) for a total of 355,028 entries which yield 16,973,937 distinct 9-mers.

MS/MS searching of PDX samples was performed against a human and mouse RefSeq database with a release date of June 29, 2018 and containing 72,908 entries.

Source data are provided with this paper in the Source Data file. The original mass spectra and the protein sequence database used for searches have been deposited in the public proteomics repository MassIVE (http://massive.ucsd.edu) and are accessible at ftp://massive.ucsd.edu/MSV000090437/. The published LUAD discovery dataset can be found on the CPTAC program website, which details program initiatives, investigators, and datasets at https://proteomics.cancer.gov/programs/cptac. Specifically, the proteomic data can be found in the public proteomics repository MassIVE (http://massive.ucsd.edu) and are accessible at ftp://massive.ucsd.edu/MSV000086793/. The genomic data can be found at the Genomic Data Commons; https://portal.gdc.cancer.gov/, via dbGaP Study Accession: phs001287.v5.p4 https://www.ncbi.nlm.nih.gov/projects/gap/cgi-bin/study.cgi?study_id=phs001287.v5.p4. The analyzed LUAD discovery sample annotations, processed and normalized data files are provided as Tables S1–S3 of the Gillette, Satpathy et al, Cell 2020 publication.

Human research participants

Policy information about studies involving human research participants and Sex and Gender in Research.

Reporting on sex and gender	n=10 LUAD patients with 4 female and 6 male. This set of LUAD samples was chosen to represent important biological differences of high relevance to lung adenocarcinoma, as five samples were driven by KRAS mutations and five by EGFR mutations. Each driver mutation subset included samples from both men and women and both Asian and Western/Caucasian ethnicity were represented. Gender was determined based on self-reporting, and it was not used for study design.
Population characteristics	This set of LUAD samples was chosen to represent important biological differences of high relevance to lung adenocarcinoma, as five samples were driven by KRAS mutations and five by EGFR mutations. Each driver mutation subset included samples from both men and women and both Asian and Western/Caucasian ethnicity were represented
Recruitment	LUAD samples were collected as part of the NIH/NCI CPTAC consortium (https://proteomics.cancer.gov/programs/cptac) with protocols mandated by the CPTAC program office. Data collection and analysis in this study was performed in accordance with the Declaration of Helsinki and Institutional review boards at tissue source sites reviewed protocols and consent documentation adhering to the CPTAC guidelines. Clinical data were obtained from tissue source sites and aggregated by an internal database called the CDR (Comprehensive Data Resource) that synchronizes with the CPTAC DCC (https://cptac-data-portal.georgetown.edu/). Clinical data can be accessed and downloaded from the DCC (Data Coordinating Center). Details about these samples have been published previously, reference: https://doi.org/10.1016/j.cell.2020.06.013. Information on participant compensation is not available to the investigators.
Ethics oversight	LUAD samples were collected as part of the NIH/NCI CPTAC consortium (https://proteomics.cancer.gov/programs/cptac) with protocols mandated by the CPTAC program office. Data collection and analysis in this study was performed in accordance with the Declaration of Helsinki and Institutional review boards at tissue source sites reviewed protocols and consent documentation adhering to the CPTAC guidelines. Clinical data were obtained from tissue source sites and aggregated by an internal database called the CDR (Comprehensive Data Resource) that synchronizes with the CPTAC DCC (https://cptac-data-portal.georgetown.edu/). Clinical data can be accessed and downloaded from the DCC (Data Coordinating Center). Details about these samples have been published previously, reference: https://doi.org/10.1016/j.cell.2020.06.013. Information on participant compensation is not available to the investigators.

Note that full information on the approval of the study protocol must also be provided in the manuscript.

Field-specific reporting

Please select the one below that is the best fit for your research. If you are not sure, read the appropriate sections before making your selection.

- [x] Life sciences
- [ ] Behavioural & social sciences
- [ ] Ecological, evolutionary & environmental sciences

For a reference copy of the document with all sections, see nature.com/documents/nr-reporting-summary-flat.pdf

Life sciences study design

All studies must disclose on these points even when the disclosure is negative.

Sample size	n=10 LUAD patient samples. The sample size was selected based on tissue availability from the NIH/NCI CPTAC consortium (https://proteomics.cancer.gov/programs/cptac). We requested tissue that had at least 100 mg available for analysis.
Data exclusions	No data exclusions were used for the 10 LUAD patients.
Replication	These finding cannot be reproduced from the 10 LUAD patients, as no additional tissue is availble for analysis.
Randomization	This is not relevant to this study.
Blinding	Details about these samples have been published previously, reference: https://doi.org/10.1016/j.cell.2020.06.013. Information on participant compensation is not available to the investigators.

Reporting for specific materials, systems and methods

We require information from authors about some types of materials, experimental systems and methods used in many studies. Here, indicate whether each material, system or method listed is relevant to your study. If you are not sure if a list item applies to your research, read the appropriate section before selecting a response.

Materials & experimental systems

n/a	Involved in the study
☐	☒ Antibodies
☐	☒ Eukaryotic cell lines
☒	☐ Palaeontology and archaeology
☐	☒ Animals and other organisms
☒	☐ Clinical data
☒	☐ Dual use research of concern

Methods

n/a	Involved in the study
☒	☐ ChIP-seq
☒	☐ Flow cytometry
☒	☐ MRI-based neuroimaging

Antibodies

Antibodies used	5 uL of K-ε-GG antibody bead slurry with proprietary antibody amounts(Cell Signaling Technology, #59322), 25 uL of PTMScan® Acetyl-Lysine Motif [Ac-K] Immunoaffinity bead slurry with proprietary antibody amounts (PTMScan® Acetyl-Lysine Motif Kit #13416), 9ug TAL-1B5 (Abcam, ab20181), 3ug EPR11226 (Abcam, ab157210), 3ug B-K27 (Abcam, ab47342), 15ug HLA-I antibody (W6/32) (Abcam, ab22432),
Validation	Only commercial antibodies were used in this study, and associated validation information can be found using the part numbers suppliers provided above.

Eukaryotic cell lines

Policy information about cell lines and Sex and Gender in Research

Cell line source(s)	A375 (ATCC)
Authentication	no authentication was used
Mycoplasma contamination	Tested negative for mycoplasma contamination
Commonly misidentified lines (See ICLAC register)	no commonly misidentified cell lines reported in ICLAC were used

Animals and other research organisms

Policy information about studies involving animals; ARRIVE guidelines recommended for reporting animal research, and Sex and Gender in Research

Laboratory animals	Patient-derived xenograft (PDX) tumors from established basal (WHIM6) and luminal (WHIM20) breast cancer subtypes were raised subcutaneously in 8-week-old NOD. Cg-Prkdcscid Il2rgtm1Wjl/SzJ mice (Jackson Labs, Bar Harbor, ME)
Wild animals	This study did not involve wild animals.
Reporting on sex	Sex was not considered for PDX study design.
Field-collected samples	This study did not involve samples collected in the field.
Ethics oversight	All experiments with live mice were performed according to institutional and national regulations and approved by the Institutional Animal Care and Use Committee at Washington University in St.Louis, MO.

Note that full information on the approval of the study protocol must also be provided in the manuscript.
